# Supplementary material for: Spectral estimation for detecting low-dimensional structure in networks using arbitrary null models
Source: PLoS One. 2021 Jul 2;16(7):e0254057. doi: 10.1371/journal.pone.0254057 (PMC8253422; doi:10.1371/journal.pone.0254057)
Supplement: S1 Appendix — (1) Demonstration of how the sparse WCM model better captures a data network’s weight distribution. (2) Results for k-partite structure in real networks. (3) Discussion of using generative null models for statistical testing of data network eigenvalues. (4) Details of the unsupervised multi-way clustering algorithm. (PDF) [file pone.0254057.s001.pdf]

Supplementary Appendix for: Spectral estimation for discovering low-dimensional structure in networks using arbitrary null models

Mark D. Humphries, Javier A. Caballero, Mat Evans, Silvia Maggi, Abhinav Singh

1 Sparse WCM captures weight distributions

Sampling from the classic weighted configuration model creates a weighted network that is likely denser than the original data network. In that model, the expectation $\langle \mathbf{P} \rangle$ defines a non-zero probability of connection between every pair of nodes, whereas real networks are predominantly sparse (Newman, 2003; Humphries and Gurney, 2008), and so the sampled weights are spread over more links than in the data network. This can create a potentially large difference in the distribution of weights between the sampled network and the real network, as we show in Fig. 1 for the Les Miserables network.

We introduce the sparse weighted configuration model (WCM) as a solution here, in which we first sample an adjacency matrix $\mathbf{A}^*$ that will be equivalently sparse to the data network on average, and then place all weights only on links in $\mathbf{A}^*$. Figure 1 shows how this sparse WCM correctly captures the weight distribution of the Les Miserables network.

2 Finding k-partite structure in real networks

For any given data network, we can equally estimate the lower bound of the eigenspectrum of $\mathbf{C}$ predicted by the null model, by taking the expectation $\langle \lambda^*_{\text{min}} \rangle$ over the minimum eigenvalues for each generated model. We can then ask if the data network has eigenvalues more negative than this predicted bound. If so, we can then retain the corresponding eigenvectors of $\mathbf{C}$, and use those to both project the network and reject nodes. The presence of large negative eigenvalues implies an approximate k-partite structure in the network, formed by groups of nodes that are more connected between the groups (and less within them) than predicted by the null models.

We find that seven of our real networks indeed had eigenvalues more extreme than the lower bound predicted by the sparse weighted configuration model. All but one had just one eigenvalue, suggesting a bipartite structure. Node rejection on the corresponding eigenvector(s) always reduced the size of the network (Fig. 2a), suggesting an embedded k-partite structure involving a sub-set of nodes.

To find the bipartite structure, we assign the retained nodes to two groups depending on the sign of their entry in the retained eigenvector (that is, positive entries to one group, and negative entries to the other). We plot examples of the resulting bipartite groups in the dialogue network of Star Wars Episode 2 (Fig. 2b), and in the adjective-noun network of the novel *David Copperfield* (Fig. 2c). Thus, applying spectral estimation to predict the lower bound of the eigenvalue spectrum can uncover k-partite structure embedded in larger networks.

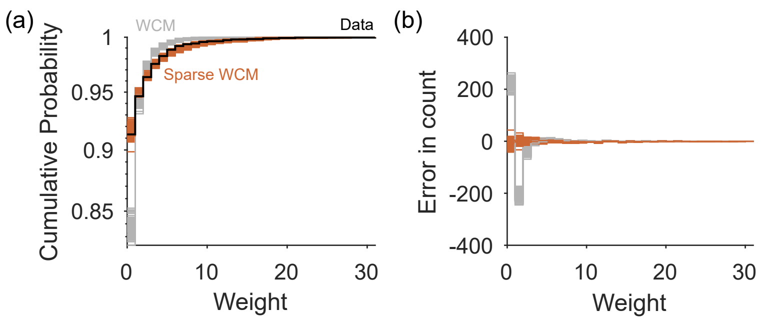

Figure 1: Network weight distributions in the null models
(a) Integer weight distribution of the Les Miserables network and generated null models. We plot the empirical cumulative distribution of the weights; one line for each of the 100 generated models of each type.
(b) Error between integer weight distributions of the Les Miserables data and null models, expressed as the difference in counts of each weight. One line per generated null model.

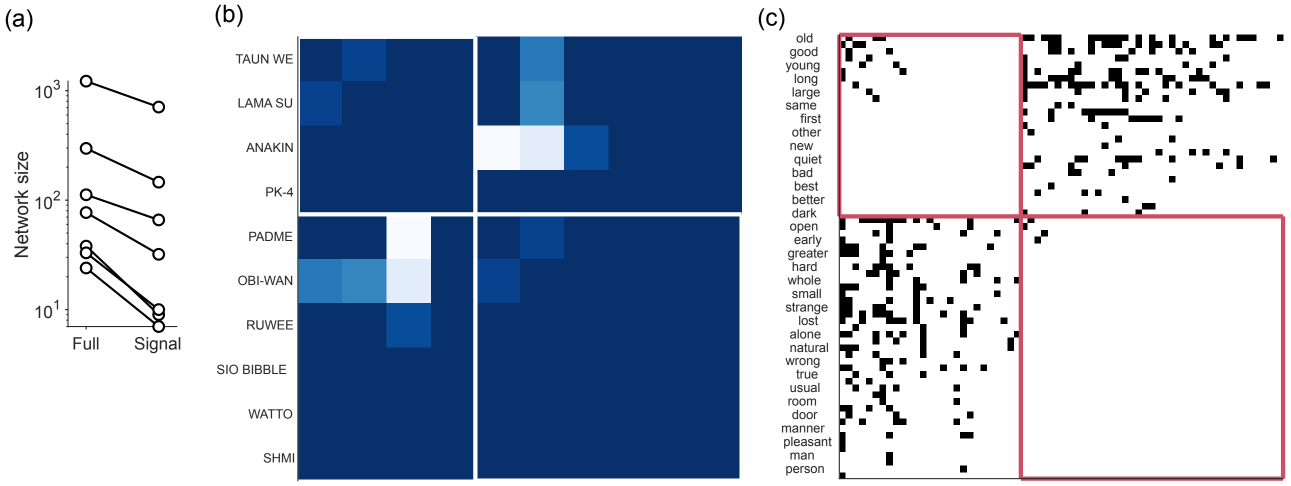

Figure 2: Details of k-partite networks
(a) Number of nodes in each full network with negative eigenvalues below the upper bound; and the number of nodes remaining after node rejection using the corresponding eigenvector(s) (for sparse WCM).
(b) Detected bipartite structure in the signal portion of the Star Wars Episode 2 dialogue network. The heatmap encodes link strength (dark-to-light $\rightarrow$ weak-to-strong) .
(c) Detected bipartite structure in the signal portion of the adjective-noun network from the novel *David Copperfield*. The network is binary, with links indicated by black entries. Here the bipartite structure is created by adjective pairs that are never found together (block diagonals), such as "young old", but which each pair frequently with other adjectives or nouns such as "strange old". Note we only label alternate nodes on the y-axis for clarity.

3 Spectral estimation as null hypothesis significance testing

In the main text we generate sampled null models to estimate the expected upper and lower bounds of an eigenvalue distribution. As a suggestion for future work, here we briefly note how one could use the same generative process to construct statistical approaches to the problem of determining whether the data network's largest eigenvalues depart from those predicted by some null model.

Every generated instance of the null model gives us an observation $\lambda^*_{\max}(i)$ of the maximum eigenvalue of such a model. One could use these in three ways to construct statistical tests of the data network's largest eigenvalues:

1. Confidence intervals. Our estimated upper bound on the null model's eigenvalue distribution is the mean $\langle \lambda^*_{\max} \rangle$ of the N maximum eigenvalues. An upper confidence interval on this mean estimate is $\langle \lambda^*_{\max} \rangle + t_{\alpha,N}\frac{S}{\sqrt{N}}$, where S is the standard deviation of the maximum eigenvalues, and $t_{\alpha,N}$ is the required percentile of the t-distribution – for example, for a 95% confidence interval, $\alpha = 0.975$. We can then compare each data network eigenvalue $\lambda_1, \lambda_2, \ldots, \lambda_n$ to this upper limit.

2. T-tests. We can test the hypothesis that eigenvalue λ_j from the data network is larger than the expectation $\langle \lambda^*_{\max} \rangle$ obtained from the null models using a one-sample, one-tailed t-test. If we define λ_j as the location parameter and $\lambda^*_{\max}(i)$ as our sample observations, then we compute the t-value $t = (\langle \lambda^*_{\max} \rangle - \lambda_j)/\frac{S}{\sqrt{N}}$, and obtain a p-value from a left-tailed t-test. In practice, we repeat for each λ_j of the data network that exceeds the expected upper bound $\langle \lambda^*_{\max} \rangle$.

3. Permutation test. Alternatively, we can test the hypothesis that eigenvalue λ_j from the data network is larger than the expectation $\langle \lambda^*_{\max} \rangle$ using a permutation test. A useful one-sample permutation test uses the test statistic $d = \sum_i \lambda^*_{\max}(i) - \lambda_j$ of the total deviations between the samples and the location parameter, then permutes the signs of the differences N times to define a p-value for d (see e.g. Chapter 3 of Good 2005). Again, we can thus obtain a p-value for each λ_j of the data network.

A script demonstrating all three approaches is in the code repository at `https://github.com/mdhumphries/NetworkNoiseRejection`. Similar approaches could be used to test the data networks smallest eigenvalues against the lower bound predicted by the null model.

4 Unsupervised multi-way vector clustering

We briefly review the multi-way clustering algorithm of (Zhang et al., 2016) using their notation. The goal is to find k communities in total. Each node has an associated vector $\mathbf{r}$ in $k-1$-dimensional space, given the $k-1$ top eigenvalues and eigenvectors of $\mathbf{C}$. For node i, vector element l is: $[\mathbf{r}_i]_l = \sqrt{\lambda_l} U_{il}$, where λ_l is the lth eigenvalue and $\mathbf{U}_l$ is the corresponding eigenvector.

Given these node vectors, the multi-way algorithm proceeds as follows:

1. Choose an initial set of group vectors $\mathbf{R}_s$, one for each of the k communities (here, chose from node vectors at random).

2. Compute the inner product $R_s^T r_i$ for all nodes i and all s sets of nodes in assigned communities, or $(R_s r_i^T) r_i$ if node i is currently assigned to community s.

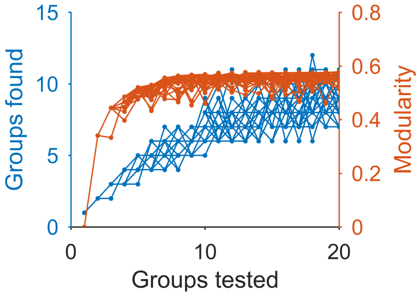

Figure 3: Multi-way spectral clustering performance on the Les Miserables network Each line is one run of the algorithm, each run is a set of partitions found using between 2 and 20 initial groups. The number of groups in the found partition (blue) plateaus later than the corresponding modularity (red) of that partition. Thus taking the maximum modularity on each run would create a wide variation in the number of groups.

3. Assign each node to the community s with which it has the greatest inner product.
4. Update the group vectors by $\mathbf{R}_s = \sum_{i \in s} r_i$
5. Repeat from step 2 until the group vectors stop changing.

In Zhang et al. (2016), the value of k for the number of communities was set by prior knowledge. In order to use multi-way spectral detection as an unsupervised algorithm, we scan k, computing the multi-way spectral partition and its modularity Q at each value of k. Here we use a maximum of $k = 20$. We could choose the value of k that maximises Q; but Q plateaus after the initial few values of k, so the choice of k can vary dramatically on different runs on the same network (Fig. 3). We solve this problem by using the location of the knee in the k vs Q curve – i.e. the start of the plateau – as the retained partition. In practice we detect this using a simple bisection procedure of fitting separate linear regressions to the values of Q either side of each k, and choosing the knee as the value of k for which the total sum-squared error of both regressions is minimised.

References

Good, P. (2005). *Permutation, Parametric and Bootstrap Tests of Hypotheses.* Springer.

Humphries, M. D. and Gurney, K. (2008). Network 'small-world-ness': A quantitative method for determining canonical network equivalence. *PLoS One*, 3:e0002051.

Newman, M. E. J. (2003). The structure and function of complex networks. *SIAM Review*, 45:167–256.

Zhang, P., Moore, C., and Newman, M. E. J. (2016). Community detection in networks with unequal groups. *Phy Rev E*, 93:012303.
