# Supplementary material for: Spectral estimation for detecting low-dimensional structure in networks using arbitrary null models
Source: PLoS One. 2021 Jul 2;16(7):e0254057. doi: 10.1371/journal.pone.0254057 (PMC8253422; doi:10.1371/journal.pone.0254057)
Supplement: Attachment — Submitted filename: Response_to_reviews.docx [file pone.0254057.s002.docx]

Dear Dr Oliva

We thank the referees for taking the time out to review our paper. All edits to the paper are highlighted in purple text within the manuscript.

Both referees raised the issue that, in their opinion, the paper was in the wrong order (that we had ordered the paper as Introduction->Results->Discussion->Methods rather than Introduction->Methods->Results->Discussion). As this was the only concern raised by Referee 1, we infer this issue was the driver of the “Major Revisions” decision.

In response to this, we refer to the Author Guidelines for PLoS One at <https://journals.plos.org/plosone/s/submission-guidelines>, which states

“The following elements can be renamed as needed and presented in any order:

- Materials and Methods
- Results
- Discussion
- Conclusions (optional)”

Papers in the format we use (Introduction->Results->Discussion->Methods) are the dominant form in many fields of science, including the default format of most general science journals (PNAS, Nature Comms, Science Advances, Scientific Reports etc). Indeed almost all papers from our lab have this format. We chose this format here because we believed it would make the ideas in the paper accessible to the broadest possible audience, which is after all why we submitted it to PLoS One.

The primary contribution of the paper is the conceptual idea of using a Monte-Carlo simulation framework to estimate the eigenspectrum expected under a chosen null model network, and then to use that estimated spectrum to quantitatively define how the given data network departs from the null model at network and node level. We use community detection as our worked example of network-level structure. Hence, the opening 4 paragraphs of the Results give, in narrative form, all of the main contributions we make, which would have been hidden had the reader had to instead wade through an extensive Methods section that by necessity focuses on the details of the simulations and models used.

**Reviewer #2:**

*1. Why the authors do not put conclusion section in this manuscript? It is hard to give any comments without it. Or do the authors have any reasons for it?*

There is no conclusion section because it is optional (see guidelines above) and we had no reason to write a separate one, having summarised the paper’s findings and our conclusions at length in the Discussion.

*2. The logical order of the manuscript is confusing. The authors have the manuscript structure as follows:*

*a) Introduction*

*b) Results*

*c) Discussion*

*d) Method*

*e) No conclusion*

Please see the general response above.

**Reviewer #3:**

*In the Introduction I miss that the authors mentioned a number of advantages (in real applications) of working with network communities rather than with the whole network. This would help to justify and motivate the proposal better.*

Community detection is a thoroughly-studied problem, with a vast literature - for example, we gave references to 5 major papers, including a 44 page review. To further motivate the focus on community detection, we add some examples of such communities, now writing on lines 14-19: “To give some examples, in social networks, these communities may be groups of friends, of collaborating scientists, or of people with similar interests; in biological networks these groups may be interacting brain regions, interacting proteins, or interacting species in a food web; more abstractly, these groups may be webpages on the same topic, or words commonly found together in English.”

*In the Introduction, why do the authors claim that N is typically 10^2 − 10^9? While likely being true, how the authors introduce a network size is a bit awkward and they need to reword it and, perhaps, add references of real-world networks.*

We simply wanted to remind the reader that networks are inherently high-dimensional, to motivate why we seek low-dimensional descriptions of them. We have reworded this phrasing on line 4 to “But the number of nodes is typically between 10^2 to 10^9” and added citations to two papers that contain details of a wide range of networks across these scales.

*In the Introduction, the authors point out the disadvantages of spectral-based community detection methods. What about other community detection methods? How do they perform?*

The referee is quite right that there are other approaches to community detection, and our paragraphs in the Introduction starting “First…” and “Second….” are about general issues with community detection methods. We can now see that the paragraph in the original manuscript beginning “Spectral approaches…” was both unclear on its main point (that spectral approaches to community detection motivated our work) and repeated material already presented (for example, on the use of null models). To address this, we have removed this paragraph, and edited the paragraph beginning “Our proposed solution” (line 40) to address that spectral approaches motivated the work.

*This is uncommon that a section about Results comes straight after the Introduction. However, such a Results section comprises a part of some theoretical background. I'm sure that the authors can propose a much better structure for the manuscript. Besides, a section with a discussion about the results also ahead of the section about the methodology development is also something that seems unfamiliar to the general structure.*

See general response above. In addition, we note that our paper structure (Introduction->Results->Discussion->Methods), rather than being “unfamiliar”, is not uncommon even to network theory papers published in PLoS One (e.g. Zhang and Zhang 2009 <https://doi.org/10.1371/journal.pone.0005686>; and the classic Rosvall and Bergstrom 2010 paper “Mapping change in large networks”: https://doi.org/10.1371/journal.pone.0008694)

*At line 417 there is suggested an improvement about the scalability of the proposal by "a future development of more efficient memory usage". Can this point be better explained? On another matter the authors mention scalability issues for networks of size over 10^5, why this number? in which cases? how can this issue be linked to the intro ranging the network sizes in 10^2 − 10^9?*

This point was simply to forewarn the reader that the bottleneck in any code implementation of the algorithms (including our current implementation) is the use of memory, not the process of generating the null models (as one might naively expect). We now give a fuller explanation of the issues in the Methods section ‘’Practical computation of the sampled null models'' (lines 592-602). For a network of 10^5 nodes, we give the explicit memory requirements for storing 100 generated null models when the data network has either real-valued, integer-valued, or binary weights (respectively 74.5 GB, 18.6GB, and 9.3GB). We have also rewritten the line (411) in the Discussion to now say networks >> 10^4, as this scale is the upper limit of those we used in the paper.

*In the generation of null models, it is not clear if it is considered links weight generation in as a process in which the links are independent to each other or an influence of the network topology may remain in the process.*

We’re not sure what aspect of the models the referee is asking about here. In both the generative null models (classic and sparse weighted configuration model), the link weights are independently drawn from a Poisson distribution. The only difference between the two models is which nodes are chosen to be linked: in the classic model, all pairs are sampled; in the sparse model, we first generate an adjacency matrix that matches the sparseness of the data network, and then we sample the link weights only for pairs of nodes linked in that generated adjacency matrix. So, for the sparse model, the “topology” of the original data network does have an influence on where the weights are placed.

*Another comment is about the perturbation over the link weights at line 459, since such a perturbation is not completely defined in the manuscript.*

This sentence was intended as a way to think about the problem as whether the data network remains within the ensemble of null-model networks consistent with the generative model. We can see though that adding further notation here was not helpful, and indeed this entire paragraph mostly reiterated the points made in the preceding paragraph - we have elected to remove it to save any confusion.

*Back to the question of independent variables or not, in the subsection "Node rejection" it is not clear if any hypothesis test shall be used to see if one node is part or it isn't of a low-dimensional structure. A bit more explanations would be welcome as well with respect to how the threshold proposed at line 518 works and it is justified.*

There is no hypothesis test described in the paper: perhaps our use of “rejection” to describe removing the nodes from the data network is the source of this misunderstanding? As noted in the main text (lines 84-85 and Figure 1) and in the Methods section “Node rejection”, the decision whether to reject a node from the data network or not is based on a comparison between its projection into the low-dimensional space and its expected projection based on the generated null models. The proposed threshold is simply that we compare the data network’s projection to its expected projection (where the expectation is computed over the individual projections of a given node in each of the generated null model networks). We interpret this as defining our boundary on whether the node in the data network is “close” to the origin. Other definitions are no doubt possible and worth exploring in future work, including tests defined by confidence intervals or null-hypothesis significance testing.

We have rewritten the section on this comparison to make clearer this definition of the threshold, and to make clearer the definition of node rejection (lines 507-508). We have also added a note that exploring other definitions of node rejection could be a fruitful extension of this work (lines 508-511)

*The subsection of "Poisson generation links" needs a better explanation on how the weights are generated. The same comment also counts for the subsection on "practical computation".*

Without specific comments on what was unclear, we have rewritten these sections to tease out the steps more carefully:

- We have added a brief explanation of the existence of real and integer valued weights, and that integer weights are equivalent to the number of links between two nodes, to lines 429-430 at the start of the Methods.
- In “Poisson generation of links” we have made the key equation for the Poisson distribution parameter more explicit (now Eq 2), and explained each of its terms immediately thereafter (lines 557-558), before returning to the original text that specifically defined each of term for each null model.
- In “Practical computation”, we have briefly explained that we convert real-valued weights to integer weights in order to use the null-models (lines 577-578). (In addition to the edits on memory detailed above)

*As a general comment, the Section of Methodology shows a collection of methods, related between themselves, but not linked in a proper framework.*

We are unclear what the referee is suggesting here. The Methods section is written in a narrative form, which links all aspects of our work together. It moves from the general idea of why we want to estimate an eigenspectra of a null model network, to the specific algorithm we propose here, through using the output of that algorithm to infer properties of the data network and its nodes. We then introduce the specific null models used in the algorithm, practical issues in its computation, and finish by outlining the details of how we tested it: synthetic networks, community detection algorithms, and the sources and types of data networks.

To make this structure clearer, we have:

- Rewritten the final paragraph of the Methods section “Spectral estimation algorithm” (lines 488-494) to more clearly state what results can be drawn directly from the output of the algorithm (and in doing so moved there material that was at the start of the immediately following “Node rejection” section).
- added a new heading structure to Methods, gathering the sections on testing of the algorithm under a new heading “Testing the spectral estimation algorithm's performance”, and moving the statements on the availability of data and code to their own section.
